# The functional connectivity of the human claustrum, according to the Human Connectome Project database

**DOI:** 10.1371/journal.pone.0298349

**Published:** 2024-04-18

**Authors:** Lluviana Rodríguez-Vidal, Sarael Alcauter, Fernando A. Barrios

**Affiliations:** Universidad Nacional Autónoma de México, Instituto de Neurobiología, Querétaro, Querétaro, México; Museo Storico della Fisica e Centro Studi e Ricerche Enrico Fermi, ITALY

## Abstract

The claustrum is an irregular and fine sheet of grey matter in the basolateral telencephalon present in almost all mammals. The claustrum has been the object of several studies using animal models and, more recently, in human beings using neuroimaging. One of the most extended cognitive processes attributed to the claustrum is the salience process, which is also related to the insular cortex. In the same way, studies with human subjects and functional magnetic resonance imaging have reported the coactivation of the claustrum/insular cortex in the integration of sensory signals. This coactivation has been reported in the left claustrum/insular cortex or in the right claustrum/insular cortex. The asymmetry has been reported in task studies and literature related to neurological disorders such as Alzheimer’s disease and schizophrenia, relating the severity of delusions with the reduction in left claustral volume. We present a functional connectivity study of the claustrum. Resting-state functional and anatomical MRI data from 100 healthy subjects were analyzed; taken from the Human Connectome Project (HCP, NIH Blueprint: The Human Connectome Project), with 2x2x2 mm^3^ voxel resolution. We hypothesize that 1) the claustrum is a node involved in different brain networks, 2) the functional connectivity pattern of the claustrum is different from the insular cortex’s pattern, and 3) the asymmetry is present in the claustrum’s functional connectivity. Our findings include at least three brain networks related to the claustrum. We found functional connectivity between the claustrum, frontoparietal network, and the default mode network as a distinctive attribute. The functional connectivity between the right claustrum with the frontoparietal network and the dorsal attention network supports the hypothesis of claustral asymmetry. These findings provide functional evidence, suggesting that the claustrum is coupled with the frontoparietal network serving together to instantiate new task states by flexibly modulating and interacting with other control and processing networks.

## Introduction

The claustrum is an irregular and fine sheet of grey matter in the basolateral telencephalon present in almost all mammals. The claustrum is separated from the insular cortex by the extreme capsule and medially from the lentiform nucleus by the external capsule [1–8]. Research in healthy subjects using diffusion tensor imaging (DTI) has revealed cortical connections with the claustrum, which possesses projections to A) prefrontal cortex, BA 8, 9, 10, 11, 12, and 34; B) visual cortex, BA 17, 18, 19 and 39; C) sensorimotor cortex, BA 7, 5, 1/2/3, 4, 6 and 8; and D) language areas BA 44, 45 and 31; as well as with orbitofrontal cortex, temporal cortex, basal ganglia and amygdala [3, 7, 8] using DTI in 100 healthy subjects, Torgerson et al. [8] found that the claustrum has the highest connectivity in the brain by regional volume. The literature about the claustrum–including studies in animal models and in humans–has evidenced the vast anatomical connections between the claustrum and the entire cerebral cortex and the subcortical structures [1–4, 6–9].

Based on its cellular composition and wide structural connectivity, the claustrum has been described as a “cornerstone of sensory integration” [1]. Similarly [2], it was proposed as a crucial component integrating motor and sensory information from different modalities to assemble them in a single experience. Early studies performed on animal models (cats and primates mainly) found evidence that the claustrum was involved in integrating sensorial stimuli. That research on the function of the claustrum was based on anatomical tracing data and electrophysiological recordings; pointing to its wide connections with almost the entire cerebral cortex, finding claustral neurons respond to visual, auditory, and somatosensory stimulation [1, 2, 4, 10–13].

Studies with human subjects and functional magnetic resonance imaging (fMRI) have reported the coactivation of the claustrum in cognitive processes such as integration of sensory signals across the tactile and visual modalities, multisensory integration of conceptually related common objects, retrieval fluency, cognitive control, and task switching [14–20].

Another extended function attributed to the claustrum outlines its role in salience processes, this could be justified not only due to the anatomical closeness with the insula but also because they share ontogeny, Pirone et al., [21] using immunohistochemistry techniques and samples of insular and temporal subunits of the human claustrum, revealed that the claustrum share ontogeny with the insular cortex, but not with putamen. Additionally, Remedios et al. [22] analyzed the single neuron recordings located in the auditory zone of the primate claustrum, concluding that the claustrum detects the occurrence of novel or salient stimuli. More recently, an fMRI study with an animal model carried out by Smith et al. [23, 24] revealed that the claustrum is functionally connected with brain areas involved in salience processes, such as the insular cortex, prefrontal cortex, and cingulate cortex [23–26]. In addition, the claustrum has been proposed as a relevant structure implicated in salience detection and attention [4, 27–29]. Furthermore, the claustrum has been involved in neurological disorders such as seizures, Alzheimer’s disease, Parkinson’s disease, schizophrenia, and disruption of consciousness [30–37]. Interestingly, claustral asymmetry was reported not only in the literature related to neurological disorders [31, 33, 34, 36, 37], but also related to cognitive processes [14–16].

On the other hand, resting-state functional connectivity (RSFC) has proved to be a useful tool to characterize the functional communication of specific brain areas. Examining RSFC allows us to determine the correlation between the spontaneous activity of brain areas that are anatomically separated, observing if there is functional communication between brain regions. Specifically, it is defined as the temporal dependency of neurophysiological events of anatomically separated brain regions [38]. To this respect, a study in humans using functional magnetic resonance imaging (fMRI) showed the functional connectivity between the claustrum and cingulate cortex, prefrontal, visual, and parietal cortices, precuneus as well as subcortical structures such as thalamus and nucleus accumbens among others; according to this study, their results suggest the association of the claustrum with cognitive control [17, 20]. Nevertheless, to have a clear or complete understanding of the function of the claustrum, we will require an accumulation of many studies to find certitude about our conclusions.

One of the most challenging issues in studying the human claustrum is its intricate anatomical location and its irregular form. Actually, the claustrum dimensions have been reported through a postmortem 3D reconstruction imaging study by Kapakin [5] (right claustrum 35.5710mm x 1.0912mm x 16.00mm and a volume of 828.8346mm^3^ and left claustrum 32.9558mm x 0.8321mm x 19.00mm and a volume of 705.8160 mm^3^) and Milardi et al. [7] have reported similar values for the claustrum mean volume. We propose approaching these challenges by assessing the claustrum’s whole brain resting state functional connectivity using the WU-Minn Human Connectome Project (HCP) dataset. The functional datasets were acquired with a spatial resolution of 2 mm isotropic and a temporal resolution of 720ms at 3T [39–43]. In this study, we aimed to explore, by a seed-driven analysis, the resting-state functional connectivity of the human claustrum based on a large cohort of healthy subjects. We expect to obtain in our result functional connectivity between the claustrum and prefrontal cortex, cingulate cortex, insula, and brain areas related to the integration of somatosensorial stimuli, and salience.

## Materials and methods

### Subjects

The study included data from 100 healthy young adults (53 females) with ages between 22 and 35 years old, representing healthy subjects who are expected to pass the age of major neurodevelopmental changes and have not onset the age of degenerative changes [41]. Subjects were randomly selected from the 1200 Subjects Data Release that is part of the WU-Minn Consortium (Principal Investigators: David Van Essen and Kamil Ugurbil; 1U54MH091657) funded by the 16 NIH Institutes and Centers that support the NIH Blueprint for Neuroscience Research; and by the McDonnell Center for Systems Neuroscience at Washington University, WU-Minn Human Connectome Project (HCP NIH Blueprint: The Human Connectome Project, https://www.humanconnectome.org/study/hcp-young-adult/data-releases) [41]. HCP excluded subjects having a history of psychiatric, neurological, or neurodevelopmental disorder or substance abuse. Van Essen et al. provide a full and detailed description of the recruitment criteria [41, 42]. Our sample from the HCP data included 100 unrelated subjects with complete resting-state functional magnetic resonance imaging (rs-fMRI) and anatomical MRI datasets. The WU-Minn HCP Consortium obtained the full informed consent from all participants following the Code of Ethics of the World Medical Association. All the protocols for data acquisition, distribution, and use of the dataset also complied with the Code of Ethics of the World Medical Association [42].

### Image acquisitions and preprocessing

The HCP acquired anatomical and resting-state functional MRI data at Washington University using a customized Siemens 3T “Connectome Skyra” with a standard 32-channel head coil. Structural dataset acquisitions included T1-weighted (T1w) and T2-weighted (T2w) images with 0.7 mm isotropic resolution, FOV = 224x224 mm, matrix = 320, 256 sagittal slices in a single slab TR = 2400 ms, TE = 2.14 ms, TI = 1000 ms, FA = 8°. Resting-state fMRI were acquired at 2 mm isotropic resolution, TR = 720ms, TE = 33.1 ms, slice thickness of 2.0 mm, 72 slices. Uğurbil et al. [43] and Glasser et al. [39] provide a full and detailed description of the HCP acquisition protocols. We include in our study the HCP dataset preprocessed by the HCP, which due to its spatial and temporal resolutions and differing distortions must be processed differently from standard neuroimaging data to achieve optimal results [39, 43]. Pipelines developed by HCP include spatial distortion correction, motion correction, spatial registration and normalization to MNI coordinates, high pass filtering (cutoff = 2,000s), and denoising of rs-fMRI data [39, 40]. In addition, we use as confound regressors BOLD signal from the white matter, cerebrospinal fluid (CSF) mask, realignment and scrubbing parameters, and band-pass filtering (0.01–0.08 Hz) [44, 45]. The time series data from regions of interest (ROIs) were not smoothed to minimize potential signal contamination from adjacent structures. The linear regression and filtering were carried out using *CONN toolbox* (V18b, Functional connectivity toolbox, NITRC) [46], SPM 12 software [47], and MATLAB R2018a (https://www.mathworks.com). The analysis used the standard resting-state pipeline in CONN toolbox to simplify eventual reproducibility.

### Mask of claustrum and functional connectivity

**Fig 1:**
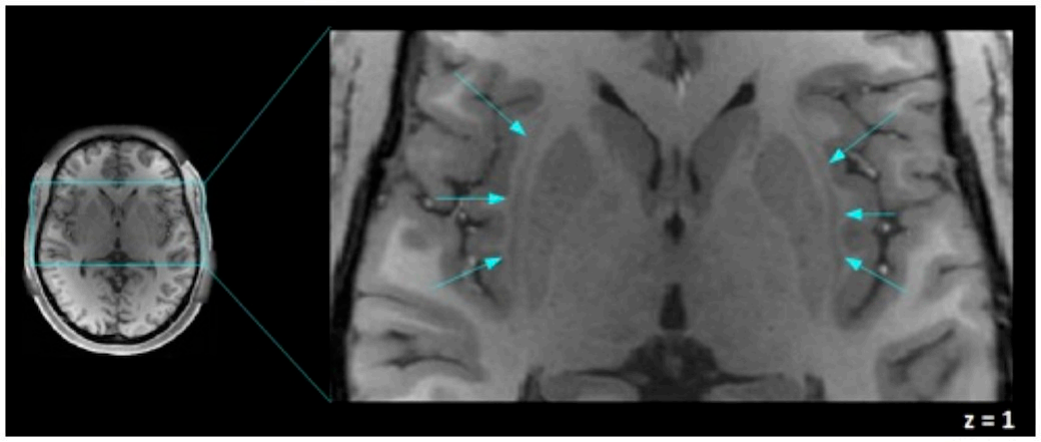
An axial view of the human claustrum. The human claustra are shown (signaled by blue arrows) between the external and extreme capsule, left and right claustra in a T1-weighted image of one of the participants are shown in MNI coordinates. This figure was acquired using Fslview, from the FMRIB software Library (FSL) tools v5.0 [48].

**Fig 2:**
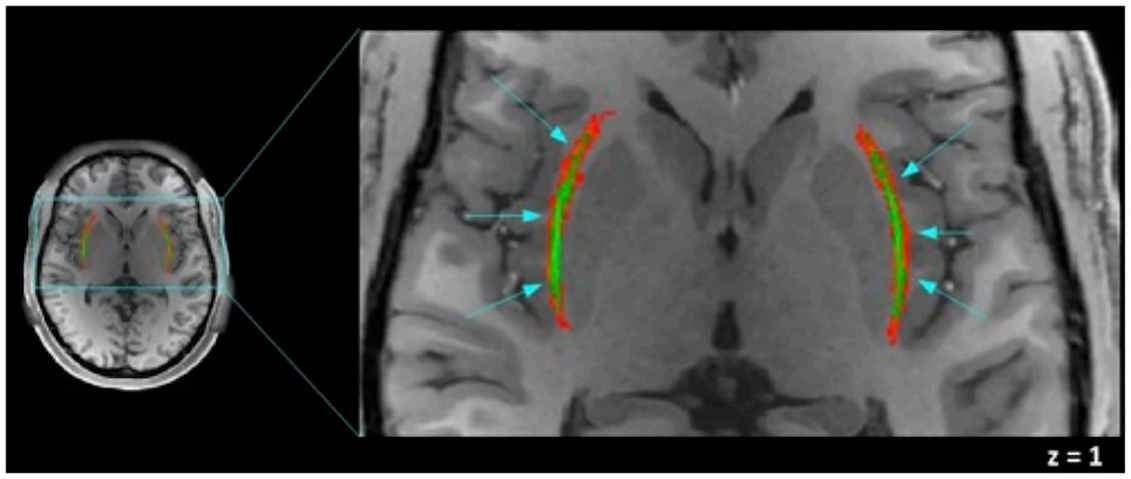
Axial view of the claustrum mask. The averaged mask of the claustrum is shown in green, left and right claustra mask on a T1-weighted image of one of the participants are shown in MNI coordinates. In green, our effective claustrum ROI, was estimated from the intersection of all subjects’ claustrum. In red, variability area due to subject differences. In our analysis we include only the green area. This figure was acquired using Fslview, from the FMRIB software Library (FSL) tools v5.0 [48].

**Fig 3:**
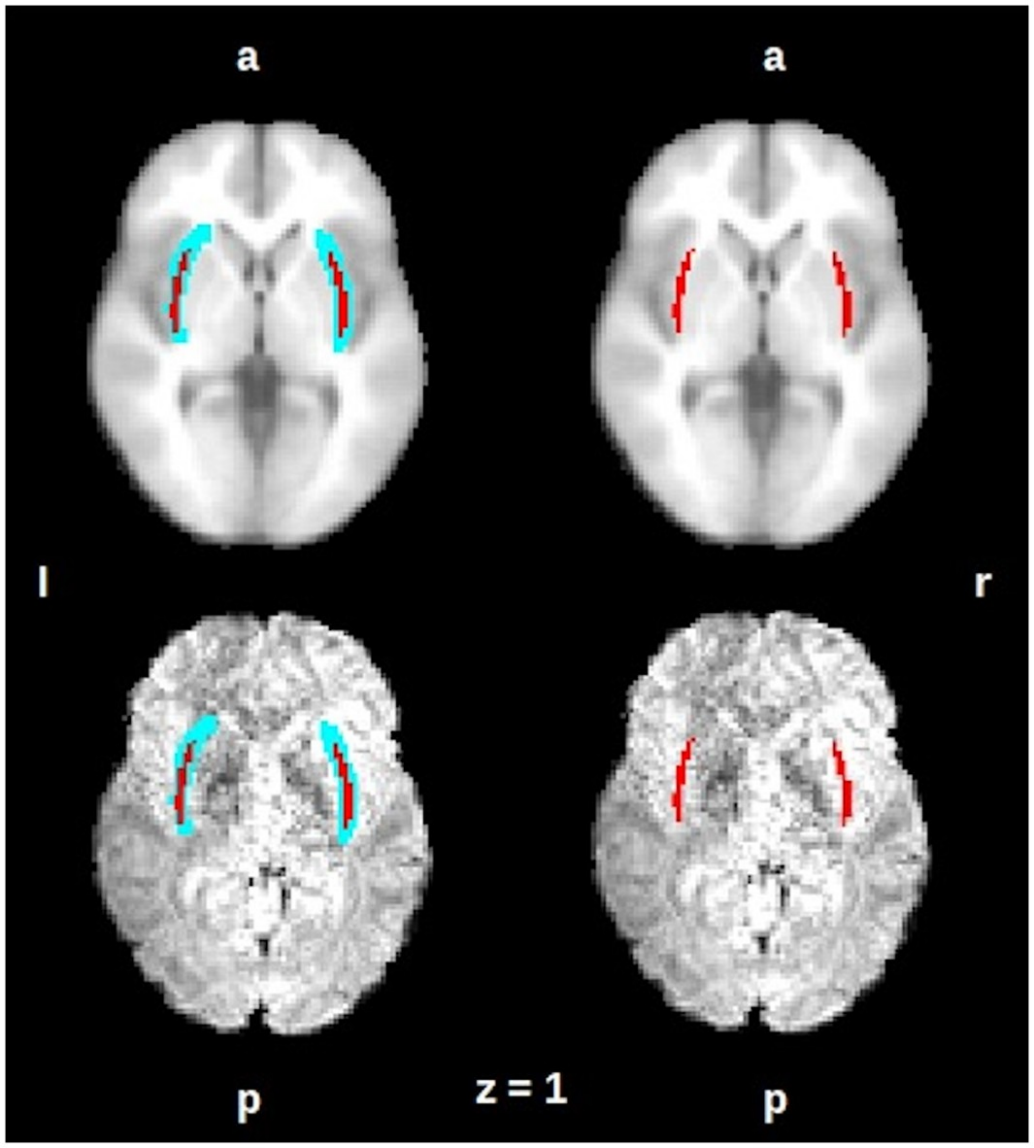
On an axial view the averaged mask of the claustrum is shown in red, left and right claustra mask on an anatomical MNI152 template (up) and on a representative subject functional image (down). The mask is shown after the transformation on the spatial functional resolution. On the left side: the blue area represents the variability area due to subject differences, on the right side, the red area represents the intersection of all subjects’ claustrum. R = right, L = left, A = anterior, P = posterior. This figure was acquired using Fslview, from the FMRIB software Library (FSL) tools v5.0 [48].

The T1 anatomical volumes were used to manually delineate right and left claustrum masks for each subject, in an axial plane, in a superior-to-inferior manner to identify the body of the claustrum and edited in the coronal and sagittal plane for accuracy (Figs 1 and 2). We transformed them into the rs-fMRI space, spatially averaged the claustrum masks and established the threshold (0.1) of the mask to transform it into a binary mask. The transformation of the mask was a linear transformation, Tri-linear interpolation method and was carried out by the Linear Image Registration Tool (FLIRT). All these processes were carried out with the FMRIB software Library (FSL) tools v5.0 [48]. The averaged claustrum masks constituted our seeds to perform a seed-based functional connectivity analysis (Fig 3).

We performed a seed-to-voxel and seed-to-ROI analysis using as seeds the right and left claustrum masks and 132 ROIs combining FSL Harvard-Oxford atlas cortical and subcortical areas and AAL atlas cerebellar areas, the analysis was carried out using CONN toolbox.

Functional connectivity maps were estimated using the CONN toolbox (V18b, Functional connectivity toolbox, NITRC) [46], SPM 12 software [47] and MATLAB R2018a. Pearson correlation coefficients were calculated within each subject in MNI space taking the seed time course and the time course of all other voxels (seed-to-voxel analysis) or the cortical/subcortical structures (seed-to-ROI analysis). The resultant correlation coefficient maps were converted to normally distributed scores using Fisher transform to permit a second-level analysis, in which, a one-sample t-test was performed. The resultant connectivity maps were corrected at map level p<0.05 using False Discovery Rate (FDR).

We carry out two comparative resting-state functional connectivity analyses of the claustrum. We contrast the insular cortex and claustrum’s functional connectivity maps and on the other hand, we compare left versus right claustrum functional connectivity maps.

## Results

The claustrum was delimited by our averaged mask, whose dimensions are 31mm x 1 mm x 17 mm., which closely coincides with the dimension described by Kapakin [5] (Figs 2 and 3); shown on an axial view. The averaged mask used in the analysis, our effective claustrum seed, was estimated from the intersection of all subjects’ claustrum.

**Fig 4:**
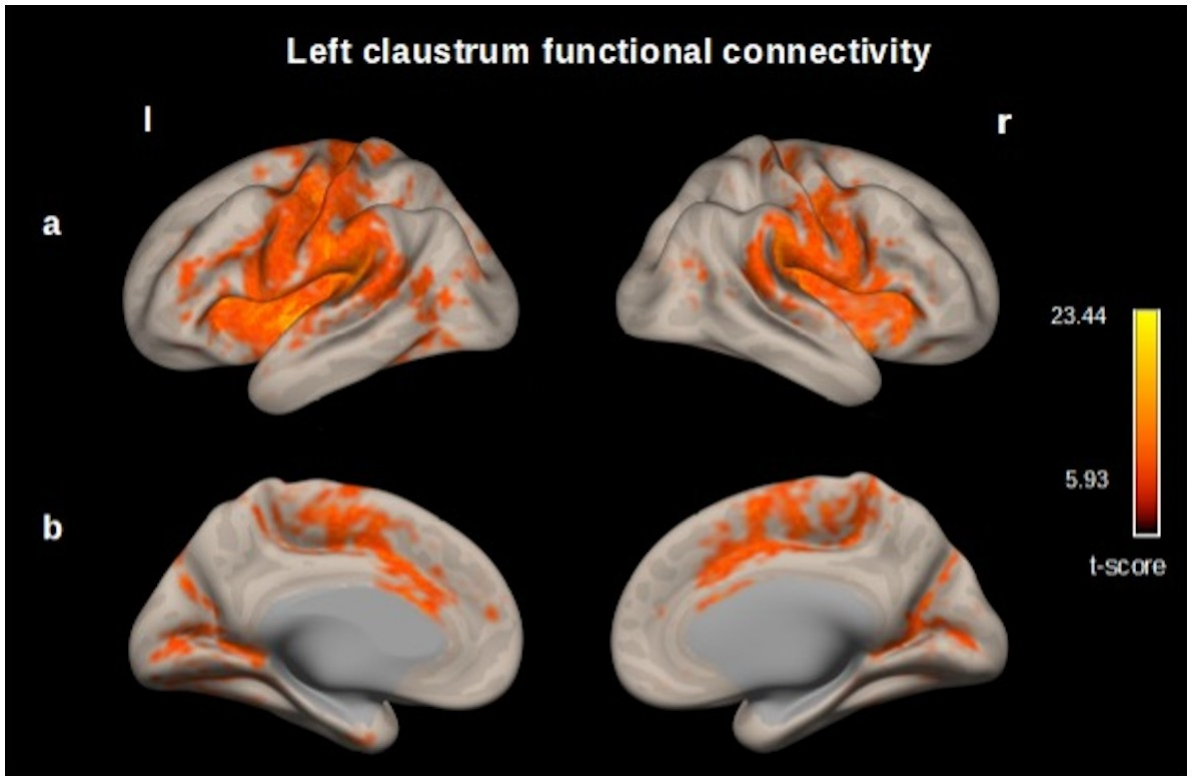
Map of the resting-state functional connectivity with left claustrum as seed. (p<0.05, p-FDR corrected) Row (A) Left, Precentral gyrus, postcentral gyrus, insular cortex, opercular cortex supramarginal gyrus. Right, insular cortex, precentral and postcentral gyrus, supramarginal gyrus. (B) Left and right, cingulate gyrus (anterior division), occipital cortex. These functional connectivity maps were estimated using the *CONN toolbox* (V18b, Functional connectivity toolbox, NITRC) [46].

In a seed-to-voxel analysis, we identified functional connectivity of the left claustrum (p<0.05 p-FDR corrected) with the following clusters: a) Left claustrum with a t–score 23.44, including left and right (l, r) precentral gyrus, postcentral gyrus (l, r), insular cortex (l, r), opercular cortex (l, r), anterior cingulate cortex, supramarginal gyrus (l, r), supplementary motor cortex (l, r), planum temporale (l, r), putamen (l, r), amygdala (l). b) Left lingual gyrus (t–score 7.92), including intracalcarine cortex (l, r), precuneous cortex, cuneal cortex (r). c) Left occipital fusiform gyrus (t–score 6.79). d) Left cuneal cortex (t–score 6.87). (Fig 4, S1 Table in S1 File).

**Fig 5:**
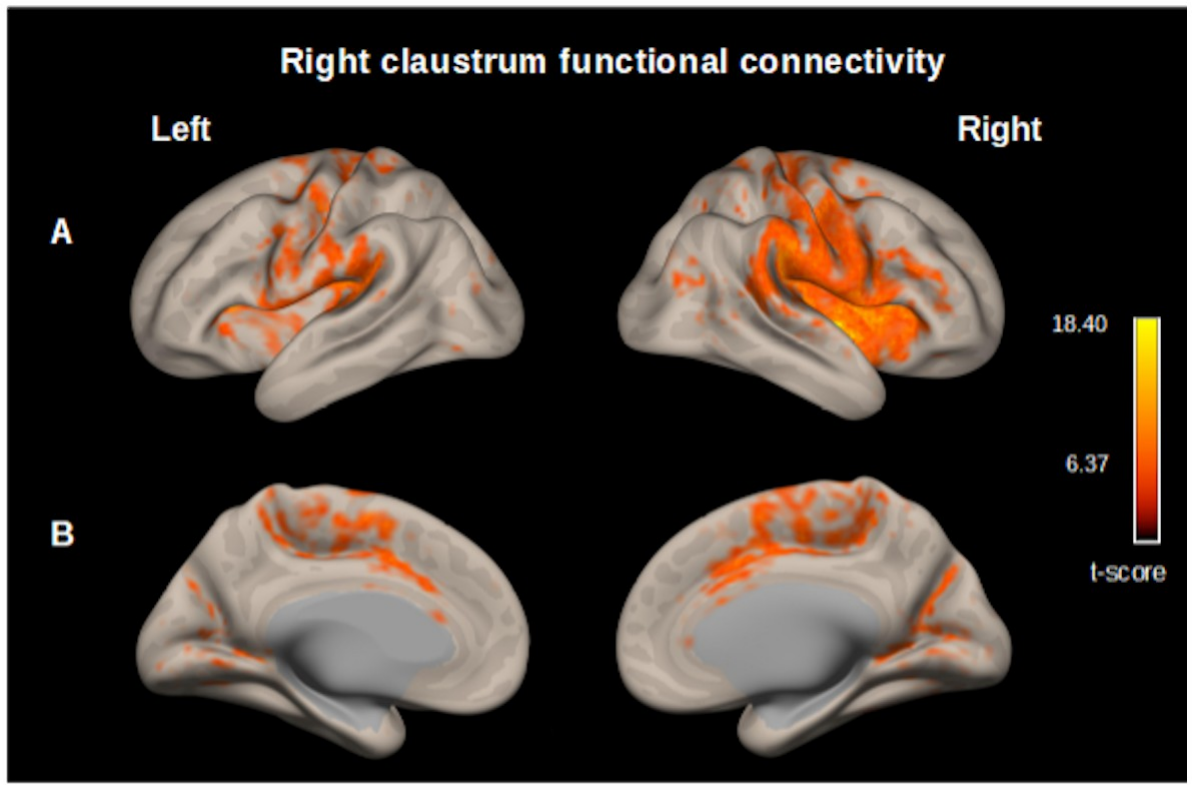
Map of the resting-state functional connectivity with right claustrum as seed. (p<0.05, p-FDR corrected) Row (A) Left, Planum polare, central opercular cortex, precentral gyrus, postcentral gyrus. Right, Precentral gyrus, postcentral gyrus, insular cortex, central opercular cortex, supramarginal gyrus, parietal operculum cortex. (B) Left and right cingulate gyrus (anterior division) occipital cortex. These functional connectivity maps were estimated using the *CONN toolbox* (V18b, Functional connectivity toolbox, NITRC) [46].

We found functional connectivity between the right claustrum and the following clusters: a) Right claustrum (t–score 18.40) including precentral gyrus (r), postcentral gyrus (r), insular cortex (r), opercular cortex (r), supramarginal gyrus (r), anterior cingulate cortex, supplementary motor cortex (r), inferior frontal gyrus (r), planum temporale (r), putamen (r), amygdala (r). b) Left planum polare (t–score 9.53) including opercular cortex (l), precentral gyrus (l), postcentral gyrus (l), insular cortex (l), planum temporale (l). c) Right lingual gyrus (t–score 7.04), precuneous cortex, intracalcarine cortex (r), cuneal cortex (r) (Fig 5, S2 Table in S1 File).

**Fig 6:**
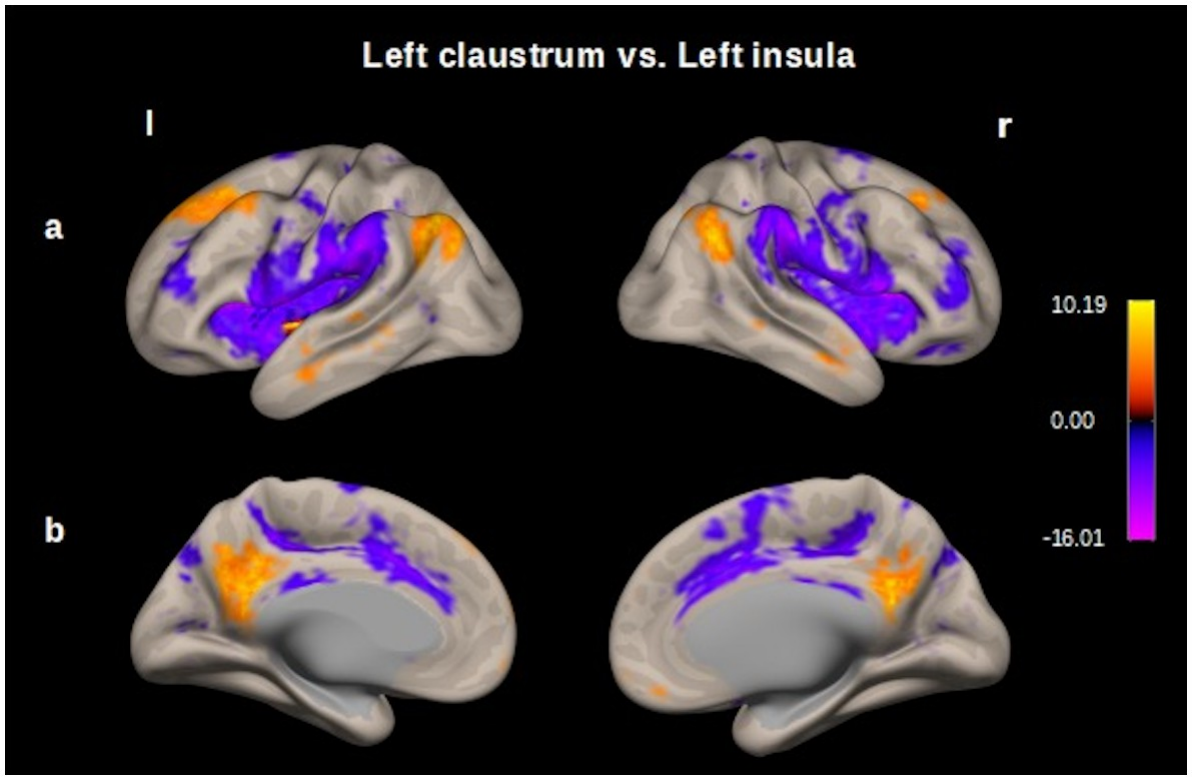
Map of the resting-state functional connectivity left claustrum vs. left insula. Seed–voxel analysis thresholded at p < 0.001 followed by p<0.05, p-FDR correction; entire sample 100 subjects. Row (A) Left and right, opercular cortex, prefrontal cortex, precentral and postcentral gyrus (functional connectivity for insula in cold colors); prefrontal cortex and posterior parietal cortex (functional connectivity for claustrum in hot colors). B) Left and right, cingulate cortex and precuneous. These functional connectivity maps were estimated using the *CONN toolbox* (V18b, Functional connectivity toolbox, NITRC) [46].

Additionally, we realized a RSFC map of the insula, which was contrasted with the RSFC map of the claustrum (claustrum > insula). We found functional connectivity between the left insula and the following cluster: a) frontal operculum cortex (l) (t–score -18.33), insular cortex (l), central opercular cortex (l), supramarginal gyrus (l), precentral gyrus (l), postcentral gyrus (l), parietal operculum cortex (l), frontal operculum cortex(l). b) planum polare (r) (t–score -16.00), insular cortex (r), central opercular cortex (r) precentral gyrus (r), supramarginal gyrus (r), parietal operculum cortex (r), inferior frontal gyrus (r), postcentral gyrus (r), frontal operculum cortex(r) and frontal orbital cortex (r). c) anterior cingulate cortex (t–score -11.77), paracingulate gyrus (r, l), supplementary motor area (r, l), superior frontal gyrus (r), posterior cingulate cortex. We found functional connectivity between the left claustrum and the following cluster: a) precuneous cortex (t–score 11.33) posterior cingulate cortex. b) lateral occipital cortex (l) (t–score 10.37), angular gyrus (l). c) superior frontal gyrus (l) (t–score 10.21), middle frontal gyrus (l), frontal pole (l). d) cerebellum crus2 (r) (t–score 9.99) cerebellum crus1 (r). e) angular gyrus (r) (t–score 9.66), lateral occipital cortex (r). (Fig 6, S3 Table in S1 File).

**Fig 7:**
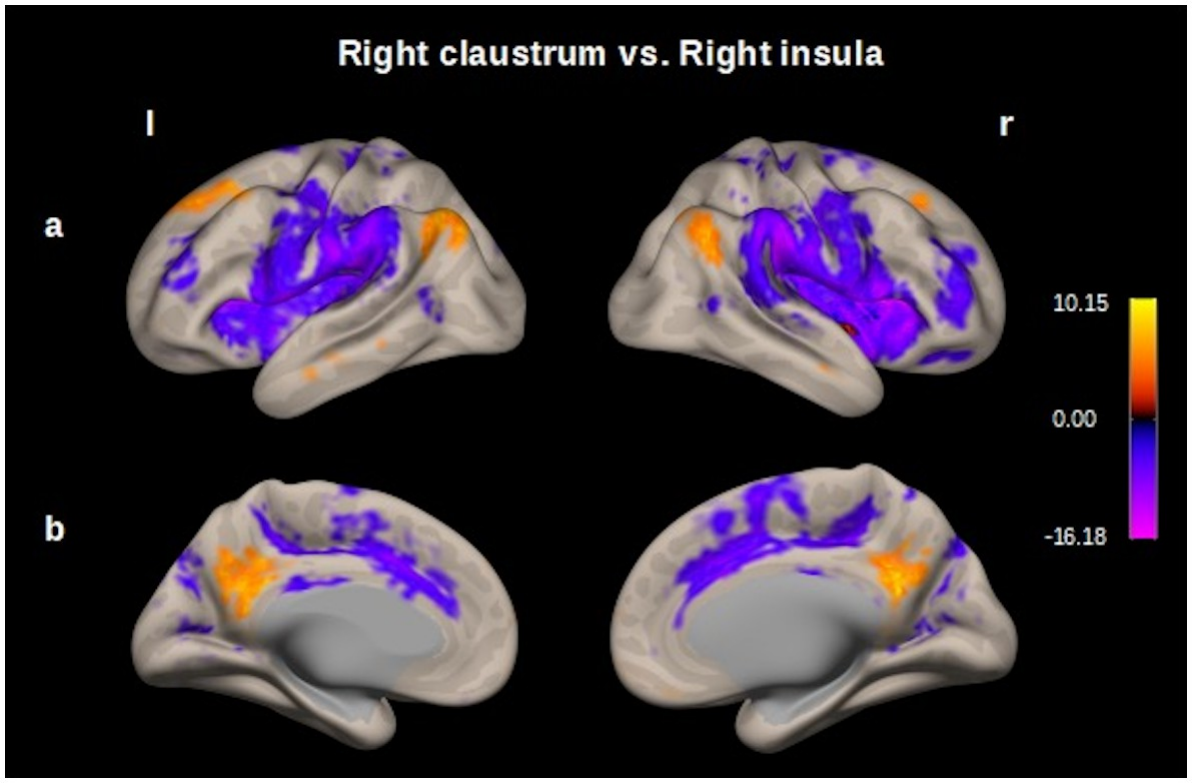
Map of the resting-state functional connectivity right claustrum vs. right insula. Seed–voxel analysis thresholded at p < 0.001 followed by p<0.05, p-FDR correction; entire sample 100 subjects. Row (A) Left and right, opercular cortex, prefrontal cortex, precentral and postcentral gyrus (functional connectivity for insula in cold colors), prefrontal cortex and posterior parietal cortex (functional connectivity for claustrum in hot colors). B) Left and right, cingulate cortex and precuneous. These functional connectivity maps were estimated using the *CONN toolbox* (V18b, Functional connectivity toolbox, NITRC) [46].

We found functional connectivity between the right insula and the following cluster: a) insular cortex (r) (t–score -17.06), precentral gyrus (r), central opercular cortex (r), supramarginal gyrus (r), postcentral gyrus (r), parietal operculum cortex (r), inferior frontal gyrus (r), frontal operculum (r), planum temporale (r), frontal orbital cortex (r). b) frontal operculum cortex (l) (t–score -17.09), precentral gyrus (l), supramarginal gyrus (l), postcentral gyrus (l), central opercular cortex (l), insular cortex (l), parietal operculum cortex (l), planum temporale (l), frontal operculum cortex (l), Heschl’s gyrus (l). c) paracingulate gyrus (l) (t–score -11.06), anterior cingulate cortex, paracingulate gyrus (r), supplementary motor cortex (r, l), superior frontal gyrus (r), posterior cingulate cortex. We found functional connectivity between the right claustrum and the following cluster: a) precuneous (t–score 11.69), posterior cingulate cortex. b) lateral occipital cortex (l) (t–score 10.16) angular gyrus (l). c) cerebellum crus2 (r) (t–score 9.83) cerebellum crus1 (r). d) cerebellum crus2 (l) (t–score 9.06), cerebellum crus1 (l). e) superior frontal gyrus (l) (t–score 9.03), middle frontal gyrus (l). (Fig 7, S4 Table in S1 File).

**Fig 8:**
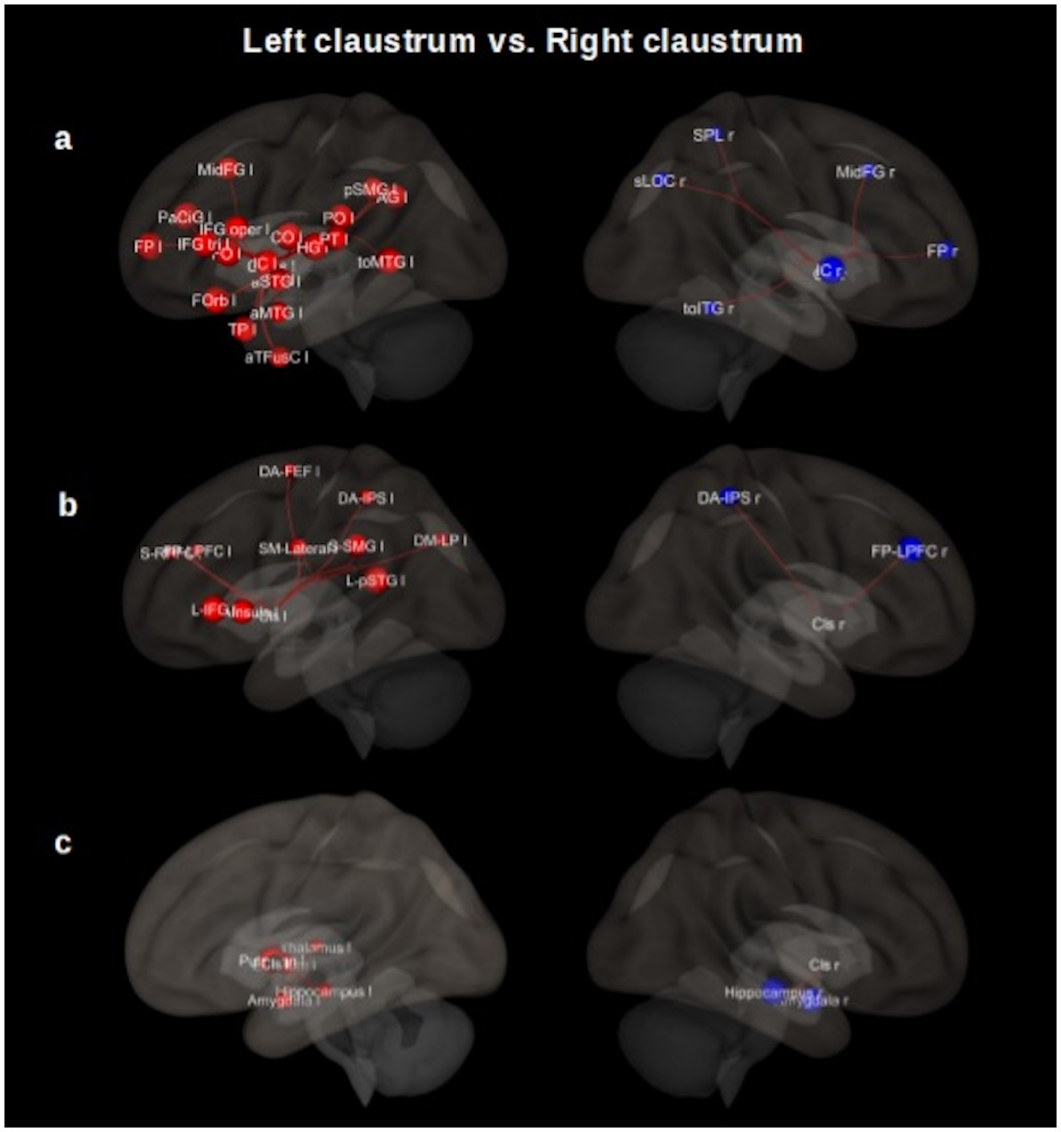
Comparison of the left claustrum with right claustrum resting-state functional connectivity (Cls–l > Cls–r). **Row (A) shows cortical areas. Left**, insular cortex (IC l), frontal orbital cortex (FOrb l), Heschl’s gyrus (HG l), frontal operculum cortex (FO l), central opercular cortex (CO l), inferior frontal gyrus (IFG tri l), paracingulate gyrus (PaCiG l), middle temporal gyrus (toMTG l), parietal operculum cortex (PO l), inferior frontal gyrus (IFG oper l), planum temporale (PT l), middle frontal gyrus (MidFG l), supramarginal gyrus (pSMG l), planum polare (PP l), superior temporal gyrus (aSTG l), superior frontal gyrus (SFG l), precentral gyrus (PreCG l), amygdala (l), supramarginal gyrus (aSMG l), superior temporal gyrus (pSTG l), postcentral gyrus (PostCG l). **Right**, insular cortex (IC r), middle frontal gyrus (MidFG r), inferior temporal gyrus (toITG r), supramarginal gyrus (pSMG r), amygdala (r). **Row (B) shows networks nodes. Left** (Cls–l > Cls–r), Language network (L): superior temporal gyrus (pSTG l), inferior frontal gyrus (IFG l). Salience network (S): insula (AI), supramarginal gyrus (SMG l), rostral prefrontal cortex (RPFC l), anterior cingulate cortex (ACC). Sensorio-motor lateral network (SM): lateral. Dorsal attention network (DA): intraparietal sulcus (IPS l), frontal eye field (FEF L). Default mode network (DM): lateral parietal (LP). Fronto–Parietal network (FP): lateral prefrontal cortex (LPFC l). **Right** (Cls–r > Cls–l), Fronto–parietal network: lateral prefrontal cortex (LPFC r). Dorsal attention: Intraparietal sulcus (IPS r). **Row (C) shows subcortical areas. Left**, putamen (l), pallidum (l), amygdala (l), hippocampus (l), thalamus (l). **Right**, hippocampus (r), amygdala (r). The results are thresholded (p< 0.05, p-FDR corrected). Cls = claustrum, l = left, r = right, a = anterior division, p = posterior division, s = superior division, tri = pars triangularis, to = temporooccipital part, oper = pars opercularis. These 3D functional connectivity maps were proyected using the *CONN toolbox* (V18b, Functional connectivity toolbox, NITRC) [46].

Left claustrum and right claustrum resting-state functional connectivity were contrasted in a seed-to-ROI analysis. When comparing pair-wise functional connectivity of the brain areas with higher connectivity values with either right or left claustrum, we obtained for left claustrum cortical areas such as insular cortex (l), frontal orbital cortex (l), opercular cortex (l) precentral gyrus (l), postcentral gyrus (l), inferior frontal gyrus (l), paracingulate gyrus (l), temporal cortex (l), putamen and amygdala. We observed a lateral predominance in the functional connectivity in the left hemisphere. We found insular cortex (r), middle frontal gyrus (r), supramarginal gyrus (r), inferior temporal cortex (r) and amygdala (r) for the right claustrum (for a complete description, see Fig 8, and S5 Table in S1 File), results were thresholded p<0.05 p-FDR corrected at seed-level.

In our contrasting analysis, we also found functional connectivity with specific nodes of networks such as superior temporal gyrus and inferior frontal gyrus (language); anterior insular cortex, supramarginal gyrus, and rostral prefrontal cortex (salience); sensorimotor lateral; intraparietal sulcus and frontal eye field (dorsal attention); lateral parietal cortex (default mode); lateral prefrontal cortex (frontoparietal) for left claustrum. Lateral prefrontal cortex (frontoparietal) and intraparietal sulcus (dorsal attention) for right claustrum. Considering subcortical regions, we obtained putamen, pallidum, amygdala, hippocampus, and thalamus for the left claustrum and hippocampus, amygdala for the right one (for a complete description, see Fig 8, S6 and S7 Tables in S1 File), results were thresholded p<0.05 p-FDR corrected at seed-level.

## Discussion

In the current study, we carried out a seed-driven resting-state functional connectivity analysis of the claustrum in a sample of 100 healthy subjects. We found that the human claustrum is widely connected with cortical and subcortical brain areas. Left and right claustrum are mainly connected with precentral gyrus, postcentral gyrus, insular cortex, opercular cortex, supplementary motor area, anterior cingulate cortex, paracingulate cortex, frontal cortex, temporal and occipital cortex, putamen, hippocampus, and amygdala. We found functional connectivity with specific nodes of well characterized brain networks such as salience (SN), sensorimotor, language, dorsal attention, default mode (DMN), and frontoparietal network (FPN) [49–52]. Our results are consistent with previous studies, which have reported functional connectivity between the claustrum and sensorimotor, parietal, temporal, prefrontal, and cingulate cortex, thalamus, and amygdala; involving the claustrum as a brain area connected with several brain networks [20, 23, 24, 26, 53, 54]. Additionally, our results show that the RSFC of the claustrum displays features of lateral asymmetry and suggests the participation of the claustrum in the frontoparietal network as a distinctive feature of the claustrum versus the insular cortex.

In line with previous studies, our findings indicate RSFC between the claustrum and cingulate cortex, insular cortex, prefrontal cortex, thalamus and amygdala, cortical, and subcortical regions related to salience processing [49, 55–57]; one of the most extended functions attributed to the claustrum [13, 22–25, 57, 58]. In the same way, our results indicate RSFC between the claustrum and motor cortex, somatosensory cortex, auditory cortex, visual cortex, amygdala and hippocampus, cortical, and subcortical areas involved with the attention processing [4, 58].

Even though the claustrum and the insula are involved in salience processes, both differ in their functional connectivity patterns when we contrasted both functional connectivity maps through a RSFC analysis. The results are consistent with the previously well-characterized RSFC pattern for the insula; however, this analysis shows functional connectivity between the claustrum and the prefrontal cortex, posterior parietal cortex and precuneus; FPN and DMN’s nodes, and cerebellum areas as a differential claustrum’s resting state functional connectivity pattern. The present results suggest that the claustrum is a node involved not only in the 1) SN, but also in the 2) FPN and 3) DMN. 1) The salience network (SN) includes the anterior insula and anterior cingulate cortex as main components. It also includes the inferior parietal cortex, lateral prefrontal cortex, amygdala, and thalamus. The SN is involved in detecting, integrating, and filtering relevant interoceptive, autonomic, and emotional information and it is considered to play a role in switching between self-awareness and the inner world mediated by the default mode network and task-related and directed attention on external stimuli carried out by the FPN. [49, 55, 56, 59, 60]. 2) Frontoparietal network (FPN) core regions are the lateral prefrontal cortex along the middle frontal gyrus (including the rostral and dorsolateral prefrontal cortex) and the posterior parietal cortex [49, 52, 59, 60]. The FPN is considered a control network due to its role in the executive, goal-directed control of information flow in the brain. Functions of this network include actively maintaining and manipulating information in working memory, decision-making in the context of goal-directed behavior, rule-based problem-solving, inhibition, and task switching. [49, 52, 56, 59, 60]. The FPN has been considered to play a role in instantiating and flexibly modulating cognitive control, this network is highly integrated with other brain networks providing functional support for rapid and flexible modulation of other brain networks [49, 52]. 3) The central nodes of the default model network (DMN) are the medial prefrontal cortex, posterior cingulate cortex, precuneus and angular gyrus [49, 56, 59, 60]. The DMN has been considered as a “task-negative” network due to it is deactivated during attention-demanding tasks. Nevertheless, it is related to different aspects of self-referential mental processes and it is active during tasks requiring autobiographical memory, prospective thinking and understanding of others’ intentions [49, 56, 59]. It has been proposed that the three networks FPN, SN, and DMN previously mentioned, which often interact and play a role in almost all cognitive functions, have been denominated as “core neurocognitive networks.” In this context, a triple-network model posits that the SN regulates interactions between the FPN and DMN. SN integrates sensory, emotional, relevant interoceptive, and cognitive information and engages the FPN brain areas that support attentional, working memory, and higher-order cognitive processes while disengaging the DMN. [59–61]. The results of the present analysis suggest that the claustrum is a common node of the core neurocognitive networks, the claustrum is not only involved in the salience process (SN) but also in instantiating and flexibly modulating cognitive control (FPN).

As an additional distinctive characteristic of the RSFC map of the claustrum, we obtained functional connectivity with the precuneus, a DMN’s node, it has been involved in autobiographical memory and understanding of others’ intentions [49, 56, 60]; in the same manner, we found RSFC between the claustrum and cerebellum crus2 that is considered as an area socially relevant due to its loop connections with key mentalizing brain areas. The cerebellum crus2 constructs sequences in advance and sends corrective feedback about different potential responses, the cerebellum facilitates spontaneous social interaction [62]. This suggests that the claustrum is related to mentalizing in some extent.

The results of our seed-driven functional connectivity analysis of the claustrum show features of lateral asymmetry in resting state healthy subjects. Functional connectivity maps of the left and right claustrum show ipsilateral and contralateral asymmetries. Nevertheless, when RSFC maps were compared, the left claustrum displayed stronger ipsilateral connectivity with superior, middle, and inferior frontal gyrus (triangularis and opercularis pars), operculum cortex (frontal, central and parietal), precentral and postcentral gyrus, posterior parietal cortex, superior temporal gyrus and middle temporal gyrus (temporo-occipital). The right claustrum displays functional connectivity with the middle frontal gyrus and posterior parietal cortex FPN’s nodes and intraparietal sulcus a node of the dorsal attention network. Claustral asymmetry has been reported before in volume [5]. In task-based fMRI research it has been reported a co-activation of the right claustrum and insula in modal sensory integration of conceptually related objects; in visual-tactile cross-modal transfer; left claustral activation in cross-modal integration of audio-visual stimuli; in response to visual-tactile integration [14–17]. In addition, a study reported decreased functional connectivity of the right claustrum with the DMN and an increased right claustrum connectivity with the FPN, an induced state by a psychedelic drug [54]. Also, the claustrum has been implicated in neurodegenerative diseases. The severity of delusions in Alzheimer’s disease and schizophrenia has been correlated with the reduction in left claustral volume [31, 33, 34, 36, 37]. Some brain areas display structural and functional hemispheric asymmetry, which appears early in development, such asymmetry may reflect specialization that supports improvements in cognitive abilities [63, 64]; for example, it is well established that the left hemisphere is dominant for language processing speech [50, 64]. The left claustrum shows ipsilateral connectivity with language network nodes (inferior frontal gyrus and posterior superior temporal gyrus). Together, the previous evidence and the present findings support the proposal for functional lateralization of the claustrum; suggesting that the left claustrum is more coupled with the brain networks previously cited, while the right claustrum is more coupled with the FPN and dorsal attention network’s nodes; serving together to instantiate new task states by flexibly modulating, interacting with other control and processing networks [17, 51, 52].

This study contributes to understanding this thin but well-connected structure: the claustrum. The use of the HCP datasets supplied us with important advantages to our study, particularly its spatial and temporal resolution; and to the standardized preprocessing accomplished by the HCP group. Nevertheless, some limitations should be noted. The age range chosen (22–35 years old) could only represent the resting state functional connectivity of the claustrum in healthy adults who are not experiencing the major neurodevelopmental changes nor neurodegenerative changes [41]; but leave out the functional connectivity claustrum in other age ranges. Our analyses are limited to the resting state and do not include task-related functional imaging. Also, we focus on presenting the connectivity of the average claustrum, we did not consider handedness or gender-related effects. We have carefully delineated a mask corresponding to the claustrum, nevertheless, this anatomical structure is itself fine, and irregular and its location complicates finer analysis. Technical constrains have limited the detailed study of the human claustrum *in vivo*; it is due to the anatomical location since it is located between two white matter structures (extreme and external capsules), irregular form taking the concavity form of the insular cortex and the convexity of the putamen, even this fine sheet-structure is not visible in some low-resolution MR imaging [12]; we consider the advantages of achieving an fMRI study with human subjects *in vivo* since most of the studies about the claustrum to the date are carried out in animal model.

In conclusion, the claustrum is a highly connected brain structure, hard to study due to its anatomical location and irregular form. Neuroimaging offers a powerful tool to explore its function non-invasively and *in vivo*, despite its limited resolution. Making use of open access datasets, we present an approximation of the resting-state functional connectivity of the claustrum, which maintains positive functional connectivity not only with cortical brain areas but also subcortical and different brain networks such as salience, default mode network, dorsal attention, and frontoparietal network. These findings provide functional evidence, suggesting that the claustrum is coupled with the core neurocognitive networks to serve together to instantiate new task states by flexibly modulating, and interacting with other control and processing networks.

## Supporting information

S1 File (DOCX)
