# Supplementary material for: The functional connectivity of the human claustrum, according to the Human Connectome Project database
Source: PLoS One. 2024 Apr 18;19(4):e0298349. doi: 10.1371/journal.pone.0298349 (PMC11025802; doi:10.1371/journal.pone.0298349)
Supplement: S1 File — (DOCX) [file pone.0298349.s001.docx]

**The functional connectivity of the human claustrum according to the Human Connectome Project database**

Lluviana Rodríguez-Vidal^1^, Sarael Alcauter^1^, Fernando A Barrios^1,^*

^1^ Universidad Nacional Autónoma de México, Instituto de Neurobiología, Querétaro, QRO, México.

**Supplementary Material**

**TABLE S1**. Left claustrum resting-state functional connectivity in the entire sample (N=100)

| **Brain Areas** | **Cluster size**  **(voxels)** | **Peak MNI**  **coordinates (mm)**  **x y z** | **Peak**  **T value** |
| --- | --- | --- | --- |
| Claustrum l/Precentral Gyrus (l)  Postcentral Gyrus (l r)  Precentral Gyrus (r)  Insular Cortex (l r)  Central Opercular Cortex (l r)  Anterior Cingulate Cortex  Parietal Operculum Cortex (l r)  Supramarginal Gyrus (l r)  Supplementary Motor Area (l r)  Planum Temporale (l r)  Putamen (l r)  Inferior Frontal Gyrus (l r)  Heschl's Gyrus (l)  Middle Frontal Gyrus (l)  Lateral Occipital Cortex (l)  Superior Frontal Gyrus (l)  Amygdala (l) | 16897 | -34 -10 +00 | 23.44 |
| Lingual Gyrus (l r)  Intracalcarine Cortex (l r)  Precuneous  Cuneal Cortex (r) | 736 | -16 -46 -04 | 7.92 |
| Occipital Fusiform Gyrus (l) | 177 | -24 -76 -08 | 6.79 |
| Cuneal Cortex (l) | 108 | -10 -78 +28 | 6.87 |
|  |  |  |  |

The results were corrected at seed level correction p<0.05 using False Discovery Rate (FDR) and were estimated using the *CONN toolbox* (V18b, Functional connectivity toolbox, NITRC) [46].

l = left, r = right

**Table S2**. Right claustrum resting-state functional connectivity in the entire sample (N=100)

| **Brain Areas** | **Cluster size**  **(voxels)** | **Peak MNI**  **coordinates (mm)**  **x y z** | **Peak T value** |
| --- | --- | --- | --- |
| Claustrum r/ Precentral Gyrus (r)  Postcentral Gyrus (r )  Insular Cortex (r)  Cental Opercular Cortex (r)  Supramarginal Gyrus (r)  Parietal Operculum Cortex (r)  Anterior Cingulate Cortex  Supplementary Motor Area (r l)  Inferior Frontal Gyrus (r)  PlanumTemporale (r)  Frontal Operculum (r)  Middle Frontal Gyrus (r)  Paracingulate Gyrus (r)  Heschl's Gyrus (r)  Superior Frontal Gyrus (r)  Putamen (r)  Cingulate Gyrus posterior  amygdala (r) | 8544 | +34 +04 -02 | 18.40 |
| Planum Polare (l)  Central Opercular Cortex (l)  Precentral Gyrus (l)  Postcentral Gyrus (l)  Parietal Operculum Cortex (l)  Insular Cortex (l)  Planum Temporale (l)  Supramarginal Gyrus (l)  Frontal Operculum Cortex (l)  Heschl's Gyrus (l) | 2173 | -40 -22 +00 | 9.53 |
| Lingual Gyrus (r)  Precuneous  Intracalcarine Cortex (r)  Cuneal Cortex (r) | 247 | +20 -56 +04 | 7.04 |

The results were corrected at seed level correction p<0.05 using False Discovery Rate (FDR) and were estimated using the *CONN toolbox* (V18b, Functional connectivity toolbox, NITRC) [46].

l = left, r = right

**Table S3**. Left claustrum and left insula resting-state functional connectivity in the entire sample (N=100)

(Left Claustrum > Left Insula)

| **Brain Areas** | **Cluster size**  **(voxels)** | **Peak MNI**  **coordinates (mm)**  **x y z** | **Peak T value** |
| --- | --- | --- | --- |
| Planum Polare (r)  Insular Cortex (r)  Central Opercular Cortex (r)  Precentral Gyrus (r)  Supramarginal Gyrus (r)  Parietal Operculum Cortex (r)  Inferior Frontal Gyrus (r)  Postcentral Gyrus (r )  Frontal Operculum cortex(r)  Frontal orbital cortex (r)  Planum Temporale (r)  Heschl's Gyrus (r) | 5026 | +42 -08 -10 | -16.00 |
| Frontal Operculum Cortex (l)  Insular Cortex (l)  Central Opercular Cortex (l)  Supramarginal Gyrus (l)  Precentral Gyrus (l)  Postcentral Gyrus (l)  Parietal Operculum Cortex (l)  Frontal Operculum cortex(l)  Inferior Frontal Gyrus (l)  Heschl's Gyrus (l)  Planum Temporale (l)  Planum Polare (l) | 4913 | -32 +20 +10 | -18.33 |
| Anterior Cingulate Cortex  Paracingulate Gyrus (r, l)  Supplementary Motor Area (r,l)  Superior Frontal Gyrus (r)  Posterior Cingulate Cortex | 2431 | -02 +14 +38 | -11.77 |
| Precuneous Cortex  Posterior Cingulate cortex | 1214 | +00 -58 +38 | 11.33 |
| Cerebellum crus2 (r)  Cerebellum crus1 (r) | 1025 | +44 -70 -42 | 9.99 |
| Lateral Occipital Cortex (l)  Angular Gyrus (l) | 1020 | -44 -76 +36 | 10.37 |
| Superior Frontal Gyrus (l)  Middle Frontal Gyrus (l)  Frontal Pole (l) | 985 | -22 +36 +48 | 10.21 |
| Frontal Pole (r) | 655 | +48 +46 +10 | -8.82 |
| Angular Gyrus (r)  Lateral Occipital Cortex (r) | 608 | +44 -56 +30 | 9.66 |

The results were corrected at seed level correction p<0.05 using False Discovery Rate (FDR) and were estimated using the *CONN toolbox* (V18b, Functional connectivity toolbox, NITRC) [46].

l = left, r = right

**Table S4**. Right claustrum and right insula resting-state functional connectivity in the entire sample (N=100)

(Right Claustrum > Right Insula)

| **Brain Areas** | **Cluster size**  **(voxels)** | **Peak MNI**  **coordinates (mm)**  **x y z** | **Peak T value** |
| --- | --- | --- | --- |
| Insular Cortex (r)  Precentral Gyrus (r)  Central Opercular Cortex (r)  Supramarginal Gyrus (r)  Postcentral Gyrus (r )  Parietal Operculum Cortex (r)  Inferior Frontal Gyrus (r)  Frontal Operculum (r)  PlanumTemporale (r)  Frontal Orbital Cortex (r)  Heschl's Gyrus (r)  Planum Polare (r)  Inferior Frontal Gyrus (r) | 7326 | +32 +24 +08 | -17.06 |
| Frontal Operculum Cortex (l)  Precentral Gyrus (l)  Supramarginal Gyrus (l)  Postcentral Gyrus (l)  Central Opercular Cortex (l)  Insular Cortex (l)  Parietal Operculum Cortex (l)  Planum Temporale (l)  Frontal Operculum Cortex (l)  Heschl's Gyrus (l)  Inferior Frontal Gyrus (l)  Planum Polare (l)  Frontal Orbital Cortex (l) | 5494 | -32 +20 +10 | -17.09 |
| Paracingulate Gyrus (l)  Anterior Cingulate Cortex  Paracingulate Gyrus (r)  Supplementary Motor Cortex (r, l)  Superior Frontal Gyrus (r)  Posterior Cingulate Cortex  Precuneous  Superior Frontal Gyrus (l) | 3219 | -02 +14 +40 | -11.06 |
| Precuneous  Posterior Cingulate Cortex | 1038 | +00 -62 +38 | 11.69 |
| Frontal Pole (r) | 834 | +30 +44 +26 | -9.26 |
| Lateral Occipital Cortex (l)  Angular Gyrus (l) | 708 | -44 -76 +38 | 10.16 |
| Cerebellum Crus2 (r)  Cerebellum Crus1 (r) | 664 | +36 -74 -38 | 9.83 |
| Cerebellum Crus2 (l)  Cerebellum Crus1 (l) | 630 | -36 -76 -36 | 9.06 |
| Frontal Pole (l) | 444 | -40 +44 +28 | -8.39 |
| Superior Frontal Gyrus (l)  Middle Frontal Gyrus (l) | 353 | -26 +22 +56 | 9.03 |

The results were corrected at seed level correction p<0.05 using False Discovery Rate (FDR) and were estimated using the *CONN toolbox* (V18b, Functional connectivity toolbox, NITRC) [46].

l = left, r = right

**Table S5.** Claustrum resting-state functional connectivity

(Cls l vs. Cls r, cortical areas as ROIs)

| **Brain Areas** | **Beta** | **T (99)** | **p-FDR** |
| --- | --- | --- | --- |
| Insular Cortex (l) | 0.20 | 10.62 | 0.000000 |
| Putamen l | 0.17 | 9.29 | 0.000000 |
| Frontal Orbital Cortex (l) | 0.12 | 8.37 | 0.000000 |
| Heschl's Gyrus (l) | 0.10 | 7.06 | 0.000000 |
| Frontal Operculum Cortex (l) | 0.10 | 6.69 | 0.000000 |
| Central Opercular Cortex (l) | 0.10 | 6.42 | 0.000000 |
| Inferior Frontal Gyrus (tri) (l) | 0.10 | 6.41 | 0.000000 |
| Paracingulate Gyrus (l) | 0.08 | 6.15 | 0.000000 |
| Middle Temporal Gyrus (to) (l) | 0.08 | 5.91 | 0.000000 |
| Parietal Operculum Cortex (l) | 0.09 | 5.75 | 0.000001 |
| Inferior Frontal Gyrus (oper) (l) | 0.09 | 5.67 | 0.000001 |
| Planum Temporale (l) | 0.08 | 5.32 | 0.000003 |
| Middle Frontal Gyrus (l) | 0.07 | 5.23 | 0.000004 |
| Supramarginal Gyrus (p) (l) | 0.07 | 4.99 | 0.000010 |
| Planum Polare (l) | 0.08 | 4.8 | 0.000022 |
| Superior Temporal Gyrus (a) (l) | 0.07 | 4.72 | 0.000028 |
| Superior Frontal Gyrus (l) | 0.07 | 4.60 | 0.000042 |
| Precentral Gyrus (l) | 0.07 | 4.50 | 0.000060 |
| Amygdala l | 0.08 | 4.28 | 0.000131 |
| Supramarginal Gyrus (a) (l) | 0.06 | 4.05 | 0.000296 |
| Superior Temporal Gyrus (p) (l) | 0.06 | 3.61 | 0.001330 |
| Postcentral Gyrus (l) | 0.06 | 3.54 | 0.001551 |
| Insular Cortex (r) | – 0.15 | – 8.93 | 0.000000 |
| Middle Frontal Gyrus (r) | – 0.05 | – 3.56 | 0.001502 |
| Inferior Temporal Gyrus (to) (r) | – 0.05 | – 3.14 | 0.005374 |
| Supramarginal Gyrus (p) (r) | -0.04 | -2.6 | 0.025362 |
| Amygdala r | -0.04 | -2.51 | 0.030775 |

The results were corrected at seed level correction p<0.05 using False Discovery Rate (FDR) and were estimated using the *CONN toolbox* (V18b, Functional connectivity toolbox, NITRC) [46].

l = left

r = right

a = anterior division

p= posterior division

s = superior division

tri= pars triangularis

to = temporooccipital part

oper = pars opercularis

**Table S6.** Claustrum resting-state functional connectivity (Cls l vs. Cls r, networks nodes as ROIs)

| Network | Brain Areas | **Beta** | **T (99)** | **p-FDR** |
| --- | --- | --- | --- | --- |
| Language | Superior Temporal Gyrus (p) (l) | 0.10 | 7.66 | 0.000000 |
| Language | Inferior Frontal Gyrus (l) | 0.10 | 6.83 | 0.000000 |
| Salience | A Insula (l) | 0.09 | 5.62 | 0.000002 |
| Salience | Supramarginal Gyrus (l) | 0.07 | 5.20 | 0.000009 |
| SensorioMotor | Lateral (l) | 0.07 | 4.31 | 0.000248 |
| Dorsal Attention | Intraparietal Sulcus (l) | 0.06 | 3.59 | 0.002322 |
| Dorsal Attention | Frontal Eye Field (l) | 0.04 | 3.12 | 0.008518 |
| Default Mode | Lateral Parietal (l) | 0.04 | 2.87 | 0.015968 |
| Salience | Rostral Prefrontal Cortex (l) | 0.04 | 2.61 | 0.028061 |
| Fronto-Parietal | Lateral Prefrontal Cortex (l) | 0.04 | 2.46 | 0.038658 |
| Fronto-Parietal | Lateral Prefrontal Cortex (r) | – 0.06 | – 4.03 | 0.000589 |
| Dorsal Attention | Intraparietal Sulcus (r) | – 0.04 | – 2.78 | 0.019125 |

The results were corrected at seed level correction p<0.05 using False Discovery Rate (FDR) and were estimated using the *CONN toolbox* (V18b, Functional connectivity toolbox, NITRC) [46].

**Table S7.** Claustrum resting-state functional connectivity (Cls l > Cls r, subcortical areas as ROIs)

| **Brain Areas** | **Beta** | **T (99)** | **p-FDR** |
| --- | --- | --- | --- |
| Putamen (l) | 0.17 | 9.29 | 0.000000 |
| Pallidum (l) | 0.08 | 4.73 | 0.000056 |
| Amygdala (l) | 0.08 | 4.28 | 0.000215 |
| Hippocampus (l) | 0.06 | 3.69 | 0.001368 |
| Thalamus (l) | 0.05 | 3.15 | 0.006453 |
| Hippocampus (r) | – 0.04 | – 2.64 | 0.023932 |
| Amygdala (r) | – 0.04 | – 2.51 | 0.026577 |

The results were corrected at seed level correction p<0.05 using False Discovery Rate (FDR) and were estimated using the *CONN toolbox* (V18b, Functional connectivity toolbox, NITRC) [46].
